# Draft genome sequence of *Acinetobacter* sp. from the nectar of the endemic Maga flower (*Thespesia grandiflora*) of Puerto Rico

**DOI:** 10.1128/mra.00448-26

**Published:** 2026-05-29

**Authors:** Ivelisse Irizarry, Christopher A. Sambolín-Pérez, Ramón E. Rivera-Vicéns, Katheryne J. Roque Cruz, Alexia S. Meléndez-Rivera, Bryan E. Rosado-Maldonado

**Affiliations:** 1 Department of Natural Sciences, Inter American University of Puerto Rico - Metropolitan Campus 19845 , San Juan, Puerto Rico; 2 Department of Sciences and Technology, Inter American University of Puerto Rico - Barranquitas Campus 19851 , Barranquitas, Puerto Rico; 3 Institute of Sustainable Biotechnology, Inter American University of Puerto Rico - Barranquitas Campus 19851 , Barranquitas, Puerto Rico; University of Manitoba, Winnipeg, Canada

**Keywords:** *Acinetobacter*, Maga flower, nectar, genome sequencing

## Abstract

Microbes in flowers play significant roles in host biology. We present the characteristics of the draft genome of *Acinetobacter* sp. isolated from the nectar of the Maga flower in Puerto Rico. This genome has a total length of 2,546,326 base pairs, a GC content of 36.28%, and 2,555 genes.

## ANNOUNCEMENT

The genus *Acinetobacter* includes species inhabiting nectar and honeybees, such as *A. nectaris*, *A. pollinis*, and *A. apis* (1–3). *Acinetobacter* sp. was isolated from the flower nectar of *Thespesia grandiflora*, an endemic tree that produces Puerto Rico’s national flower, the Maga flower. Nectar- and bee-associated *Acinetobacter* species have undergone genomic reduction compared with other genus members (4, 5). Adaptation to the nectar environment may drive genomic streamlining in these lineages. This isolate was sequenced because it originates from an endemic plant, allowing comparison with similar nectar-associated *Acinetobacter* from other hosts. The genome of this isolate of *Acinetobacter* sp. was sequenced to describe its characteristics and composition.

*Acinetobacter* sp. was isolated in January 2025 from nectar collected with a micropipette in San Juan, Puerto Rico (18°20′18.5″N 66°04′10.1″W). It was isolated using serial dilutions and incubating on potato dextrose agar (PDA, Millipore) at 30°C for 24 hours. Preliminary identification using the 16S rDNA sequence (PZ234510.1) showed 99.6% similarity to *Acinetobacter* ESL0695 (CP113932.1) from stingless bees and 99.6% to *Acinetobacter nectaris* SAP 970.1 (JQ771134.1) from nectar.

Genomic DNA was extracted using the GenElute Bacterial Genomic DNA Kit (Sigma-Aldrich). DNA concentration and purity were evaluated with a NanoDrop One spectrophotometer (Thermo Fisher Scientific). The DNA was purified using AMPure XP beads (Beckman Coulter). The library was prepared using the Native Barcoding Sequencing Kit V14 (Oxford Nanopore Technologies) and sequenced on PromethION (Oxford Nanopore Technologies) (6). All manufacturers’ protocols were followed without modification. Basecalling was performed using Dorado v1.0.2 (7). Raw reads were quality-trimmed using fastp v1.0.1 (8), assembled with Flye v2.9.5 (9, 10), polished with Medaka v2.2.0, and annotated with Prokka v1.14.6 (11, 12). Assembly features were assessed using QUAST v5.3.0 (13). Assembly contamination and completeness were evaluated using CheckM v1.2.4 (c__Gammaproteobacteria UID4201) (14), and BUSCO v6.0.0 (c__gammaproteobacteria_odb10) (15). Prophage regions were identified using Phigaro v2.4.0 (16). Default software parameters were used in all instances. Average nucleotide identity (ANI) was calculated using FastANI v1.34 (17) using the *Acinetobacter* ESL0695 reference genome. A genomic map was generated using GenoVi v0.2.3 (18).

**Fig 1:**
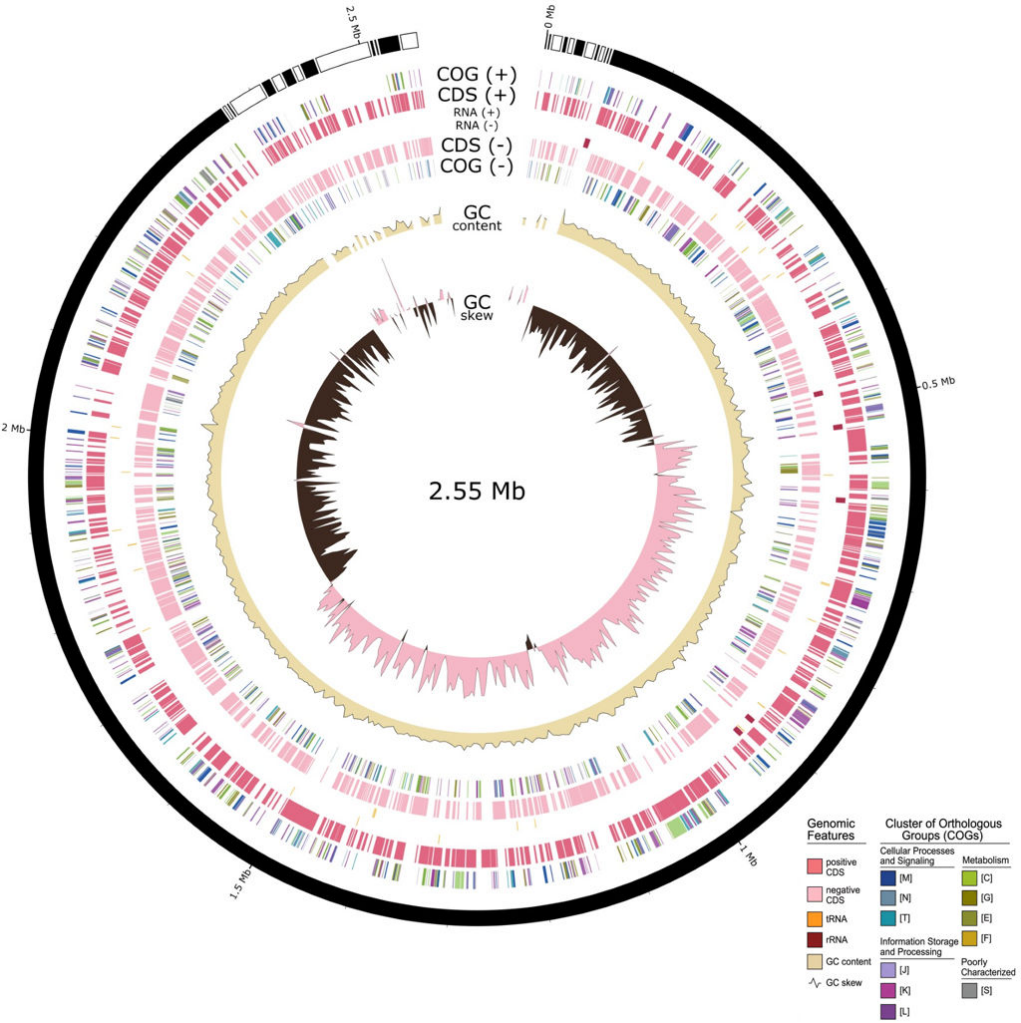
Circular genome map of Acinetobacter sp. (~2.5 Mb). Rings represent clusters of orthologous groups (COGs), functional categories, coding sequences (CDS; positive and negative strands), RNA genes (tRNA and rRNA), GC content, and GC skew. Selected COG categories highlight biologically relevant functional groups across the genome. These include cellular processes and signaling, metabolism, information and storage processing, and poorly characterized COGs.

The sequencing run produced 268,705 reads, totaling 1,291,871,883 bases, an N50 of 10,021 bp, and a sequencing depth coverage of 507×. The genome of *Acinetobacter* sp. was assembled into a single chromosome from 25 contigs, resulting in 2,546,326 bp. QUAST analysis showed an assembly N50 of 2.3 Mb, indicating high contiguity. The chromosome contains 2,555 predicted genes: 2,459 protein-coding, 78 tRNA, 18 rRNA genes, with a GC content of 36.28% (Fig. 1). CheckM and BUSCO showed 100% and 97.8% completeness, respectively. Phigaro analysis found three prophage regions totaling 86,556 bp, encoding 124 genes. ANI of the assembled genome was 84.41% with *Acinetobacter* ESL0695 as reference.

## Data Availability

The genome sequence of *Acinetobacter* sp. is available in GenBank under BioProject PRJNA1447535, BioSample SAMN57037554, accession number JBWXUO000000000, and contigs JBWXUO010000001–JBWXUO010000025. The raw sequences are available in the SRA under accession number SRR38144259.
